# StandUPTV: A full-factorial optimization trial to reduce sedentary screen time among adults

**DOI:** 10.21203/rs.3.rs-5984168/v1

**Published:** 2025-02-24

**Authors:** Sarah Keadle, Kristina Hasanaj, Krista S. Leonard-Corzo, Arlene Fernandez, Lena Freid, Skylar Weiss, Maria Legato, Harsh Anand, Todd Hagobian, Siobhan Phillips, Suzanne Phelan, Kate Guastaferro, Ryan Seltzer, Matthew Buman

**Affiliations:** California Polytechnic State University; Arizona State University; Arizona State University; Arizona State University; California Polytechnic State University; California Polytechnic State University; California Polytechnic State University; Arizona State University; California Polytechnic State University; Northwestern University; California Polytechnic State University; New York University; Arizona State University; Arizona State University

**Keywords:** sedentary behavior, television viewing, physical activity, multiphase optimization trial, mHealth application, screen time

## Abstract

**Background:**

Using the multiphase optimization strategy (MOST) framework, we aimed to identify a feasible, acceptable and optimized set of mHealth-delivered behavioral strategies for reducing recreational sedentary screen time (rSST) by at least 60 min/day.

**Methods:**

Eligible participants were 23–64 years old and had high rSST (> 3 h/day). We used a full factorial (23) design in which participants received a “core” mHealth application and were randomized to combinations of three components (on vs. off): LOCKOUT: rSST electronically restricted; TEXT: rSST reduction prompts; and EARN: rSST through physical activity. rSST was assessed at baseline and at 8 and 16 weeks of age via an integrated measure of sedentary time and screen time. We used a linear mixed effect model to test the change in rSST for the three intervention components and their interactions.

**Results:**

A total of 82% of the randomized participants (N = 110) were female, with a mean ± SD age of 41 ± 11.7 y and a BMI of 29.7 ± 7.8 kg/m^2,^ and their mean (95% CI) rSST was 184.7 (172.8, 196.5) min/day at baseline. The expected difference (baseline vs 16 weeks) in rSST was greatest for the intervention versions with a core, LOCKOUT, TEXT, & EARN (−125.7 [−172.0, −79.3] min/day) at the “on” level. The participants were satisfied with the study and found the app helpful in reducing rSST (> 94%). Technical issues resulted in 20% being somewhat dissatisfied with the app.

**Conclusions:**

We identified several promising intervention versions that exceeded our optimization objective. The intervention version that included core, LOCKOUT, TEXT, & EARN components “on” was efficacious, feasible and acceptable and should be used to test the effect of rSST reductions on health outcomes.

**Trial registration:**

(clinicaltrials.gov NCT04464993)

## Background

Television viewing consumes ~ 55% of discretionary time, an average of over 3 hours/day. (1, 2) The widespread adoption of social media, video games, and streaming services that can be accessed via smartphones and tablets has resulted in alarming net increases in total recreational sedentary screen time (rSST). (1–3) This increasing trend was exacerbated by the COVID-19 pandemic. (4–6) Newer methods of consuming rSST also promote ‘binge viewing’ (i.e., watching consecutive episodes in a continuous bout) (7), which is particularly harmful to health. (8) There are consistent and robust associations between prolonged TV viewing and numerous poor health outcomes, including diabetes, cardiovascular disease, mental health and mortality. (9–14) The risk of poor health is greater for TV viewing than for other sedentary behaviors and the the negative effects of TV viewing are not fully eliminated through engaging in recommended amounts of physical activity. (15–17) Emerging evidence suggestions that non-TV forms of rSST (social media and video games) are also associated with poor sleep quality, mood, and less physical activity. (18–20)

Given the high prevalence of rSST and the known health risks, reducing the time spent on rSST is an important target for public health, yet few intervention studies have been conducted to reduce rSST among adults. In a 2016 systematic review of behavioral strategies to reduce screen time in adults (21) conducted by the Community Preventive Services Task Force, only two studies were identified. (22, 23) Since that time, we have been aware of just one additional intervention that targeted screen time among adults.(24, 25) Collectively, the available studies all reported significant reductions in SSTs of > 60 min/d but were limited by small samples (N < 40), short durations (3–8 weeks), and only traditional TV viewing. Furthermore, no study has compared the combined effect of multiple strategies to reduce SST, and none has adapted to contemporary rSST consumption (i.e., social media, tablets, streaming media), which precludes recommendations on optimal intervention strategies to reduce rSST for adults.(21)

In the present study, we applied the multiphase optimization strategy (MOST) framework, which consists of three phases (preparation, optimization and evaluation) designed to efficiently and systemically develop and evaluate multicomponent interventions. (26) In the previously published preparation phase, we identified three candidate components with an optimization objective to carry forward the smallest number of components that achieve a reduction in the rSST of at least 60 minutes while considering the user burden and acceptability of the intervention package (i.e., all active components optimization objective). (27) The present paper includes the results from the optimization phase where we conducted a 16-week a 2^3^ full- factorial randomized trial yielding eight experimental conditions that evaluated the effects of the three candidate components, alone and in combination, on total rSST. We also present the results for feasibility, acceptability and user burden as well as secondary outcomes of total sedentary time and MVPA.

## Methods

### Participants

The trial was registered at clinicaltrials.gov (NCT04464993). Recruitment occurred at two study sites (ASU and Cal Poly), and the full study protocol has been published previously. (27) Data collection was scheduled to begin in July 2020 but was delayed until May 2021 because of COVID-19. Specifically, we removed planned cardiovascular biomarker assessment, and the protocol was modified to conduct all assessments virtually, which opened recruitment to those outside the Phoenix, AZ and San Luis Obispo areas but delayed the start of the study. Enrollment continued until April 2023, and data collection was completed in September 2023. Recruitment methods included newspaper advertisements, flyers on campus and throughout the local community, ResearchMatch.com, social media (i.e., Facebook, Twitter) and other social networking sites, and the sending of electronic newsletters and fliers to local businesses.

Eligible participants were between 23 and 64 years of age because of their high levels and heterogeneous media consumption and elevated risk for developing chronic diseases. Enrollment and randomization were stratified by age group (23– 44.9 y vs. 45– 63.9 y). The eligibility criteria were described in detail previously. (27) Briefly, participants self-reported > 3 h/day sedentary screen time, < 3 on the Stanford Leisure-Time Activity Categorical Item (L-Cat), a brief physical activity screener (28), had no medical contraindications for MVPA, had smartphone and WiFi access and were willing to comply with the study procedures. In response to difficulties with recruitment during the COVID-19 pandemic and because in-person visits with biomarkers were removed from the protocol, we lowered the BMI threshold to include those with a BMI ≥ 18.5 kg/m2 on April 11, 2022. The screening forms were completed via Research Electronic Data Capture (REDCap). Eligible individuals received an email with the online version of the informed consent document that was approved by the Arizona State University Institutional Review Board (IRB #00012109) and scheduled a video conferencing visit with staff who explained the study procedures and expectations of the study participants. Eligible and interested participants electronically signed the informed consent document online via REDCap. (29, 30)

#### Enrollment and baseline procedures

After providing consent, the participants were mailed via U.S. Postal Service a technology kit that included several devices for tracking rSST: a Wi-Fi plug per television in the home (WeMo Insight Smart Plug); one Wi-Fi router; one Raspberry Pi (i.e., a small computer to record and transmit data); one Samsung Galaxy tablet preloaded with apps that participants stated they regularly used on their smartphones; and a Fitbit Charge 4. The participants were encouraged to use the tablet for all screen time that was not otherwise consumed on their home television(s). Baseline assessments were performed over 7 days. All screen time was monitored, and participants were able to use the study tablet but did not yet have access to intervention content or feedback on rSST. The participants were required to have ≥ 4 days of measured screen time (i.e., verification from the study server that tablet and WeMo data were transmitted > 10 h/day) during the baseline period and were asked to repeat the baseline test if they had < 4 days recorded.

#### Randomization and Intervention

The participants were randomized into one of 8 experimental conditions representing all combinations of the three candidate components and levels (Table 1) – stratified by age category (23.0 to 44.9y vs. 45.0 to 64.9y) - via the MOST randomization module in RedCap. (31) Following the baseline assessment, participants attended a virtual visit with a study staff member where they were notified of their experimental condition, reviewed their motivations for joining the study and reasons for wanting to reduce their rSST and a brief orientation to the app that included reviewing one educational lesson of their choosing and demonstrating how to navigate the app, including features specific to the components in their assigned experimental condition. Investigators were blinded to intervention assignment.

#### Intervention

The component selection and intervention details were previously published and are briefly described here. All participants received a core intervention via the StandUPTV mHealth application that included (a) a target to reduce screen time by 50% compared with their baseline values; (b) rSST self-monitoring tools (i.e., the gauge, graphs and charts); and (c) 16 lessons, including education and behavioral change content.

##### LOCKOUT.

Screen-limiting features were enabled for both the traditional TV (via the WiFi plug) and the recreational apps on the tablet when rSST reached the prescribed threshold (50% of baseline) within a week-long period. The 50% baseline allotment was reinstated at the beginning of the next week. Participants were granted one “mulligan” per week that enabled them to delay a lockout by 60 minutes (e.g., to finish a show). Participants also received a planning tool interface for scheduling screen time up to one week in advance.

##### TEXT.

Participants with the TEXT condition on received between 1–3 prompts per day, which included (a) encouraging use of the intervention strategies; (b) 7-day rSST summaries; (c) summaries of specific rSST behaviors (e.g., “You have watched X hours of screen time over the last 7 days in bouts of 1 hour or longer”); and (d) encouraging prompts regarding rSST and (e) adaptive contextual messages on the basis of time of day and screen time (e.g., “Time to hit pause on screen time! Get up and dive into something new!”).

##### EARN.

Participants were able to earn screen time by engaging in Fitbit-assessed MVPA in 10-minute bouts at a ratio of 1:3 (i.e., 10-min exercise earns 30-min rSST). Additionally, those with EARN and LOCKOUT were able to earn more screen time to avoid LOCKOUT. The gauge display was modified for this component to emphasize the overall weekly rSST goal, time spent in MVPA, and earning balance (i.e., the difference between total screen time and amount earned). They also received six additional educational lessons regarding MVPA (e.g., safe exercising, barriers to exercise) and an interactive interface to plan MVPA time.

#### Measures

The assessment timepoints were baseline, mid-intervention (8 weeks), and postintervention (16 weeks). We focus on 16-week outcomes only to remain consistent with our optimization objective.

Primary outcome: rSST was assessed using a time-synchronized combination of accelerometer-measured sedentary time (activPAL) and screen time data from the StandUPTV mHealth application. The technical details have been previously published and are briefly described below. (27)

Posture and activity intensity were assessed using the activPAL3c micro accelerometer (PAL Technologies Ltd, Glasgow, Scotland). The devices were waterproofed using a medical grade adhesive covering and attached to the midline of the thigh with breathable, hypoallergenic tape, enabling continuous wear for consecutive days without removing for bathing or other water-based activities. Participants were asked to wear the device 24 hours a day for 7 days, and the following periods were excluded: (a) continuous sitting or standing behavior > 6 hr (considered nonwear); (b) days with ≤ 10 hours of valid wear time during the wake period; and (c) participants with only 3 or fewer valid days of activPAL wear. The data were processed into events of sitting/lying, standing, or stepping using the activPAL software version 7.2.37. All wake time measured by the activPAL as lying/seated was categorized as sedentary. Stepping time was split into periods of light-intensity physical activity ([LPA]; <100 steps/minute) and MVPA (≥ 100 steps/minute). (27)

Screen time was assessed via a combination of direct measurement from WiFi Plugs to monitor television power state (Wemo Insight, Belkin, El Segundo, CA) and tablet app usage (Samsung Galaxy, Samsung). All television was considered recreational. The study staff categorized more than 730 tablet apps, such as video games, television/video, social media, or nonrecreational.

The three streams of data (TV, tablet, activPAL) were merged at the minute level for the primary determination of rSST. To label a minute as rSST, it was required to be both sedentary by the activPAL and recreational screen time by either the tablet or television. Users were able to add or modify screen time bouts through the app when screen time occurred outside of the home or on alternative devices or reject bouts when another household member was viewing the screen.

Feasibility was assessed by examining retention metrics, including formal withdrawal from the study and completed assessments at 16 weeks.

Acceptability We used a combination of quantitative and qualitative approaches to assess acceptability in an exit interview conducted after the 16-week assessment. The participants rated overall satisfaction with the StandUPTV app (i.e., 5-item Likert scale), how often they used it, whether they would continue to use the app or recommend it to others, and whether it was helpful in reducing rSST. We also examined satisfaction with staff and technical support. Additional questions were asked that were relevant to specific components (e.g., those with TEXT on were asked to rate how helpful the text messages were in changing behavior). The response options were scored from 0–10, with 0–2 considered “not at all”, 3–7 considered “mostly” and 8–10 considered “very”. In addition, qualitative exit interviews were conducted among all participants following the 16-week assessment period via a series of open-ended questions.

User burden was assessed on the User Burden Scale, a 20-item scale that measures burden across six domains (difficulty of use, physical, time/social, mental/emotional, privacy, financial).(32) The highest possible score is 80; domain scores are categorized as low (0–0.15; < 15th percentile), somewhat low (0.2–0.45; 16–35th percentile), moderate (0.5–1.2; 36th–85th percentile), moderately high (1.25–1.7; 86th–94th percentile) and high burden (1.75–4; 95th + percentile).

### Sample size and statistical analysis

We aimed to enroll 240 participants to achieve a final sample of 200 (20% dropout; n = 25 per condition), which afforded power = .80 to detect main effects for each of the intervention components (LOCKOUT, TEXT, and EARN) and the resulting 2- and 3-way interactions, assuming a balanced experimental design and a modest baseline-to-postintervention correlation (r = .3) and α = .05. (33) We powered the study on a Cohen’s d effect size = 0.4, representing a 60 min/day reduction in sedentary behavior. This effect size was conservative, as previous rSST trials reported effects d > 1.0.(22–24) Owing to delays in initiating and slowing recruitment during the COVID-19 pandemic, we did not meet the enrollment targets. Trial concluded when funding ended.

Multiple imputation using chained equations (MICE) was employed to address missing data due to dropout, nonresponse, or incomplete data collection using the MI procedure. The imputation process was performed in 25 iterations.(34, 35) After imputation, the analyses were conducted using the pooled estimates from the multiple imputed datasets. We used a linear mixed model (proc MIXED) to test hypotheses regarding the main effects of three candidate intervention components and their interactions on the primary outcome, rSST. Effect coding was where components were coded as 1 when on and − 1 when off, and we evaluated all single component (e.g., LOCKOUT*time) two- and three-way interactions (e.g., LOCKOUT*EARN*TEXT*time) within the model. Notably, in the full-factorial design, conditions are assigned orthogonally, and estimation of each main effect and interaction effect utilizes data contributed by all the subjects in the trial. For example, the interpretation of the intervention version (also referred to as intervention component combinations) that includes CORE & TEXT is a comparison of experimental conditions 1, 2, 3, and 4 (where TEXT was “on”) versus experimental conditions 5, 6, 7, and 8 (where TEXT was “off”) (Table 1). The standard errors and confidence intervals were adjusted for the variability between imputations via Rubin’s rules and the MIANALYZE procedure.(36) These analyses, including imputation procedures, were repeated for secondary outcomes, including sedentary time and MVPA. All analyses were conducted in SAS 9.4.

Feasibility and acceptability outcomes were assessed using qualitative and quantitative data. The quantitative data were analyzed via descriptive statistics, whereas the qualitative responses were analyzed via an applied rapid qualitative analysis approach.(37) The user burden scale was summarized using descriptive statistics (e.g., means, standard deviations, frequency) overall and by subscale: 1) difficulty of use; 2) physical; 3) time and social; 4) mental and emotional; 5) private; and 6) financial. The responses were categorized based on previous research as low burden by percentile scores (16th–35th), moderate burden (36th–85th), moderately high burden (86th–94th) and high burden (≥ 95th). (32)

## Results

**Figure 1:**
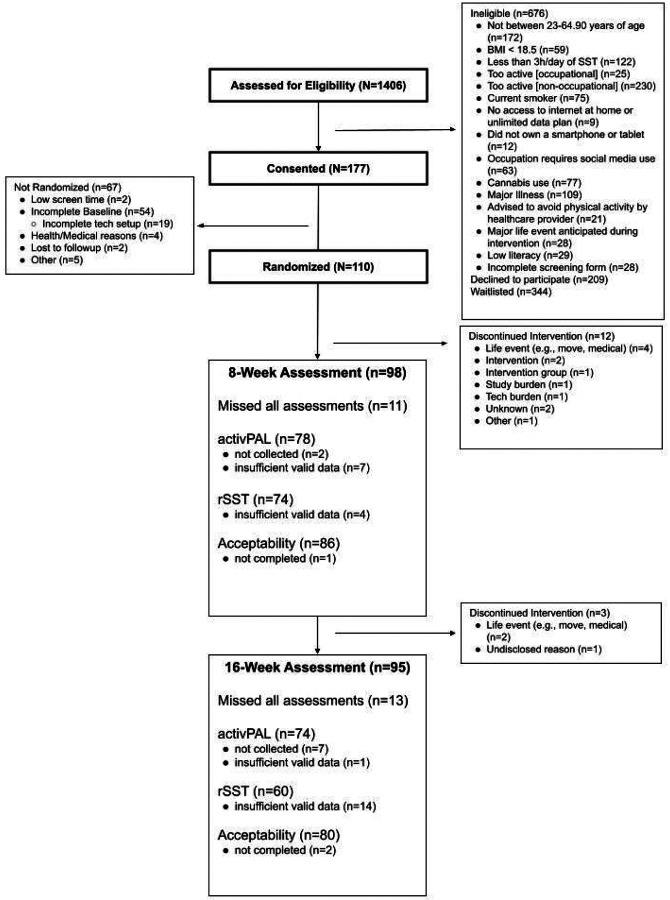
Consort Diagram illustrating flow of study participants.

Figure 1 presents the participant flow for recruitment, randomization, and retention. In total, 1,406 individuals were assessed for eligibility; 177 consented, and 110 were randomized. A total of 95 participants completed the 16-week intervention (86% retention), with the majority of those who discontinued the intervention doing so within the first 8 weeks and due to life events (e.g., relocation or medical). The participant demographics and baseline study outcomes are presented in Table 2. The sample was predominantly female, middle-aged, overweight, and non-Hispanic White (22% Hispanic). At baseline, participants engaged in > 3 hours of rSST (measured objectively via activPAL and the screen-time measurement platform) and > 10 hours of total sedentary time (measured via activPAL) per day. There were no appreciable differences in baseline characteristics or outcome measures at the component level.

### Intervention impact on rSST at 16 weeks

**Figure 2:**
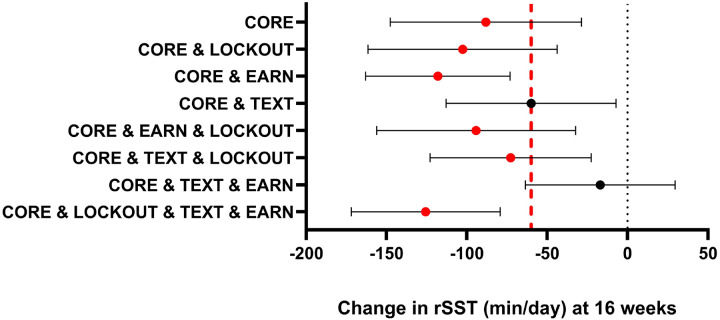
Expected outcomes (Change in rSST) by Intervention Version Note: Values are min/day.

At 16 weeks, 75% of the participants provided data for analysis. The rSST and overall sedentary time outcome data were missing, primarily because of insufficient valid data from the activPAL or technical errors in the screen-time monitoring platform. There was an overall significant main effect of time, as participants engaged in a mean (95% CI) of 184.7 (172.8, 196.5) min/day of rSST at baseline, which was reduced to 99.8 (85.6, 114.0) min/day at week 16. The expected difference (baseline vs 16 weeks) in the primary optimization outcome (rSST) is shown in Fig. 2 for each intervention version and in Table 3 for the mean at each time point. The expected difference exceeded the *a priori* identified optimization objective of 60 min/day for 6 of the 8 intervention versions. The two intervention versions with the greatest expected differences were EARN & TEXT & LOCKOUT (−125.7 [−172.0, −79.3] min/day) and EARN (−118.1 [−163.0, −73.1] min/day). The additional intervention versions exceeding 60 min/day were LOCKOUT (−102.7 [−161.5, −43.8] min/day), TEXT & LOCKOUT (−72.8 [−122.8, −22.6] min/day), and EARN & LOCKOUT (−94.3 [−156.2, −32.4) min/day]. The only intervention version with some indication of antagonism among components was TEXT & EARN, with a reduction in rSST of −15.9 (−23.8, −8.0) min/day, and TEXT met but did not exceed the optimization objective (−60.1 [−113.0, −28.8] min/day).

#### Secondary outcomes: impact on sedentary time and physical activity.

There was no significant reduction in total sedentary time at 16 weeks (Table 3) and no evidence that any component led to reductions in total sedentary time. Overall, there was no significant increase in MVPA in any of the intervention versions.

### Study feasibility, user burden, and acceptability

Table 4 shows the feasibility, acceptability and user burden overall and by component. Among the 110 randomized participants, 11 withdrew (10%) for reasons related to the study (e.g., burden), an additional 5 (4.5%) withdrew for unrelated reasons (1 moved out of the country, 1 moved and started school, 2 reported unrelated injuries, 1 no longer had a stable living situation), and 14 were unresponsive at the 16-week assessment (12.7%). This resulted in 80/110 (72.7%) randomized participants completing the 16-week assessment. Two harms were reported that were deemed unrelated to the intervention: severe concussion and undisclosed injury. Both participants withdrew. One participant withdrew due to reported mild skin irritation caused by the Fitbit. Retention was slightly higher when LOCKOUT was off vs on (76.4% vs 69.1%) and higher when EARN was on (77.2%) vs off (69.8%). **Supplemental Table 1 **shows the same results across the experimental conditions; retention was highest among LOCKOUT & TEXT and EARN & TEXT & EARN (> 85%) and lowest among LOCKOUT & EARN and LOCKOUT & TEXT (< 55%), but the given small cells should be interpreted with caution.

The acceptability scores are shown in Table 4. Overall, and across components, participants were very satisfied with the study staff (> 98.5% satisfied or very satisfied). They were satisfied with technical support from staff (84.8%), although satisfaction was slightly lower when LOCKOUT was on (74.6%) than when LOCKOUT was off (92.1%). The mean (SD) user burden score was 7.2 [6.5] on a scale ranging from 0–80 (**Supplemental Table 1**). The highest subdomain score was for *difficulty of use*, which was 0.8 (0.6) and was considered a moderate burden. *The scores for the time, social, mental, emotional and privacy* domains were all somewhat low, and the scores for the *financial* and *physical* domains were very low. Burden scores did not vary by condition.

In response to the question “rate your satisfaction with the StandUPTV app,” 54.5% reported being somewhat or very satisfied, 25.6% were neutral, and 19.7% were somewhat or very dissatisfied. Interestingly, despite modest overall satisfaction, a high proportion said that StandUPTV was helpful in reducing rSST was (93.4%), that they would recommend the app to other people (91.8%), would download a similar app to their smartphone (83.9%) and would be interested in continuing to use the app after the study was over (78.7%). The majority (60.7%) used the app at least daily. The quantitative responses for the component-specific questions are shown in **Supplemental Table 2**. When TEXT was on, a greater proportion of the participants were not satisfied with the app (18.8%), and the majority (53%) did not find the text messages helpful. As noted in **Supplemental Table 3 **with the qualitative themes, several people reported not receiving or missing notifications. Some had issues with multiple notifications appearing at once and reported swiping away all the notifications. Feedback on the content and frequency of messages was evenly distributed among those who found it not at all, mostly and very helpful. Those who liked the messages responded to the personalized messages in response to screen time and those that they perceived as tailored to their interests. The majority of the EARN participants stated that the opportunity to earn screen time through exercise was very helpful (69.4%). They were more satisfied with the feedback on exercise (55.6% very satisfied) than with the feedback on the planning tool (11.1% very satisfied). The majority reported that the earnings ratio was adequate or too high, and several stated that it could be lowered to 1:2.

The qualitative feedback is summarized in **Supplemental Table 4**. In response to the question “What did you like least about the StandUPTV app?”, the feedback was primarily related to the tablet and the bout verification process used to authorize screen time. The most well-liked feature they was the self-monitoring gauge that tracked weekly screen time, which participants reported increased their self-awareness of their rSST and was a simple way to track screen time across platforms. They noted awareness of their total amount of free time and how much of that was consumed by screens.

## Discussion

We identified several promising intervention versions that exceeded our optimization objective. The core intervention consisted of self-monitoring, a target reduction of 50% and behavior change content. The two intervention versions, in addition to the core, that produced the greatest expected differences were EARN alone and LOCKOUT & EARN & TEXT. Both intervention versions had comparable feasibility, retention and acceptability scores, and there are pros and cons in both intervention versions. EARN is a parsimonious single component, while the multi-component version of LOCKOUT & EARN & TEXT resulted in nominally greater expected outcomes without additional user burden. In making a final optimization decision between these two components we selected LOCKOUT & EARN & TEXT based on data that providing consequences for not adhering to the target behavior (i.e., LOCKOUT), and timely and relevant TEXT messages have both been previously identified as key components of successful campaigns to improve health behaviors and may maximize the longer-term behavior necessary for a successful evaluation trial. Notably, there is a need for additional refinement to improve usability and reduce the number of technical errors experienced when the TEXT was “on” in this trial.

The results of our intervention are similar to those of previous studies. Otten et al. reported a > 2 hour/day reduction in self-reported SST using an electronic “lockout” method after 8 weeks. (22) Participants were given a feasible and acceptable weekly target of a 50% reduction in SST, and when the limit was reached, the individuals’ TVs were shut off until the following week. Similarly, Raynor et al. demonstrated a > 2 hour/day reduction in self-reported SST by instructing adults to gradually decrease TV watching via behavioral strategies such as stimulus control (e.g., removing remote control) and preplanning in combination with electronic monitoring of television (but not lockout) during a 3-week intervention period.(23) Our two intervention versions with the greatest expected difference also produced a difference of 2 hours/day.

Although there were significant reductions in rSST, there were no significant changes in total sedentary time or MVPA. Targeted changes in rSST did not have substantive spillover effects on other behaviors; more specifically, participants appeared to replace rSST with nonrSST sedentary behaviors rather than physical activity. Because we were focused on rSST, we purposely did not include physical activity promotion materials in the CORE and suggested a range of “alternative activities” that include both physical activities and sedentary behaviors. Indeed, despite some targeted messaging in the EARN condition around physical activity, the results for MVPA changes were equivocal across intervention versions. Our results are broadly consistent with those of the Raynor study (23), which revealed that a “decrease TV” alone did not result in increases in steps or MVPA, but a group that received both “decrease TV plus increase PA” did show improvements in steps and MVPA. While there may be health benefits for replacing rSST with other sedentary behaviors, given the known benefits of MVPA, it would be ideal to leverage reductions in rSST into increases in physical activity. (38) Collectively, these findings reinforce that intervention approaches that can simultaneously target both behaviors are needed.

Compared with the core component, the TEXT component did not result in greater reductions in rSST in several of the intervention versions and was antagonistic in combination with EARN. This is somewhat surprising, as TEXT messages are effective in increasing physical activity. (39) We believe that there are technological and conceptual issues that help explain why this component was not as successful in this study. The message came to the user’s tablet as app notifications as opposed to SMS messages received on a smartphone, and some participants in the exit survey expressed frustration that multiple notifications sometimes came through at once when they logged into the tablet and that it was difficult to return to older messages. Research suggests that “irrelevant” messages have a negative impact on physical activity (40). In this study, most of the messages were contextual (i.e., targeted at a specific time of day or triggered by the length of the current rSST bout); if the messages were not received at the intended time due to technical issues and were thus deemed “irrelevant”, it may explain the lower rSST reductions and dissatisfaction with this component for those individuals. The MOST framework is iterative, enabling movement back to earlier phases for refinement. (26) It is critical that the TEXT condition is refined to ensure message delivery is timely and relevant, both of which are key components of successful TEXT campaigns to improve health behaviors,

Based on technological challenges and participant feedback, there are other important lessons learned that will inform the refinement of future studies. Several participants reported that they viewed the educational content at the beginning and not throughout the intervention period; providing weekly lessons may enhance continued engagement with important behavior change content. The participants overwhelmingly preferred having the app on their own smartphone rather than using a study tablet. Presently, this is possible for android phones and not for those using iOS, without additional permission through Apple, and we plan to evaluate these approaches in future studies. Several participants noted that the earnings ratio was too high (i.e., it was too easy to earn rSST), and future studies should examine whether ratios should be reduced (e.g., 2:1) or personalized. Addressing these technical issues is expected to enhance the acceptability and efficacy of the app in future iterations.

This study has important strengths and directly addresses several research gaps from systematic reviews of sedentary behavior interventions among adults. (21, 25, 41) We included a broad age range that includes distinct generations (millennials through baby boomers) who have distinct rSST consumption habits. The StandUPTV app is a scalable mHealth intervention that requires minimal staff and participant interaction after randomization. This study was longer and included a larger sample than previous rSST studies did, and our factorial design enabled the testing of individual and combined effects of components. In contrast to previous studies that relied on self-report questionnaires as the primary outcome measure, we used a novel objective measure that includes multiple components of rSST beyond just TV viewing, moving this research area into the modern era of rSST assessment. There are important limitations to this study. As described above, the use of the study tablet negatively impacted participant satisfaction and intervention fidelity. Future research needs to ensure that the application can be installed on participants’ existing devices and include smartphone measures. Because the device was not downloaded on smartphones, participants were instructed to manually input bouts of smartphone use into the app, and for the latter half of the study, we requested that participants upload daily screenshots of their smartphone’s reported daily app usage and total screen time (i.e., screen time feature on Apple, digital well-being feature on Samsung) during the assessment weeks. The technology burden for setup was relatively high and resulted in a greater than anticipated number of dropouts in the baseline period, and strategies to increase engagement and adherence to assessment protocols in a virtual study should be employed to increase response rates. Future studies should examine alternate WiFi plugs and associated technology to minimize subject burden. Although the study is substantially larger than previous rSST interventions in adults, delays in study start and difficulties in recruitment meant that we did not achieve our initial recruitment goal of 240. We also ended up with a sample with a high proportion of women, and recruitment strategies to ensure a greater and more balanced sample of men are needed in future studies. Finally, although the present study was conducted at least twice as long as previous studies did, future studies should examine whether behavior change is sustained beyond 16 weeks.

## Conclusions

StandUPTV is the first known study to systematically evaluate different strategies to reduce rSST among adults. We found that the greatest reduction in rSST for intervention versions that included EARN & LOCKOUT &TEXT components combined with the core resulted in an mHealth intervention that was efficacious, feasible and acceptable. Given that several efficacious strategies to reduce rSST have been identified, future research should examine whether strategies are equally as effective in sustaining behavior change. This study provides important evidence on the optimal intervention package to move forward to the evaluation phase of the MOST framework to test the effect of rSST reductions on health outcomes.

## Figures and Tables

**Table 1:** Experimental Design

Experimental condition	Core (educational materials, 50% target, self-monitoring)	TEXT	LOCKOUT	EARN
1	ON	ON	ON	ON
2	ON	ON	ON	OFF
3	ON	ON	OFF	ON
4	ON	ON	OFF	OFF
5	ON	OFF	ON	ON
6	ON	OFF	ON	OFF
7	ON	OFF	OFF	ON
8	ON	OFF	OFF	OFF

**Table 2:** Baseline demographics and outcome measures by component level.

	Sample Size	Female No. (%)	Age M (SD) years	BMI Mean (SD) kg/m2	Race, No. (%)				Ethnicity, No. (%)		rSST M (SD) min/day	Total sedentary time M (SD) min/day	MVPA M (SD) min/day
					White	AA	Asian	Other	Not Hispanic	Hispanic			
Overall	n = 110	90 (81.8%)	42.0 (11.7)	29.7 (7.8)	82 (74.6%)	5 (4.6%)	14 (12.7%)	12 (10.9%)	87 (79.1%)	23 (20.9%)	184.1 (125.8)	641.4 (98.8)	20.6 (14.8)
LOCKOUT													
On	n = 55	46 (83.6%)	42.3 (11.8)	31.4 (9.1)	41 (74.6%)	3 (5.5%)	6 (10.9%)	7 (12.7%)	44 (80.0%)	11 (20.0%)	193.5 (122.1)	646.4 (105.3)	19.9 (17.1)
Off	n = 55	44 (80.0%)	41.7 (11.7)	28.1 (5.9)	41 (74.6%)	2 (3.6%)	8 (14.6%)	5 (9.1%)	43 (78.2%)	12 (21.8%)	175.5 (129.8)	636.9 (93.2)	21.1 (12.4)
TEXT													
On	n = 59	47 (79.7%)	41.6 (12.1)	28.9 (6.3)	41 (69.5%)	3 (5.1%)	8 (13.6%)	6 (10.2%)	46 (78.0%)	13 (22.0%)	167.4 (105.3)	640.0 (104.6)	22.2 (16.9)
Off	n = 51	43 (84.3%)	42.4 (11.3)	30.7 (9.3)	41 (80.4%)	2 (3.9%)	6 (11.8%)	6 (11.8%)	41 (80.4%)	10 (19.6%)	205.8 (146.8)	643.2 (92.0)	18.5 (11.2)
EARN													
On	n = 57	45 (79.0%)	40.8 (11.8)	28.8 (7.4)	39 (68.4%)	3 (5.3%)	11 (19.3%)	4 (7.0%)	47 (82.5%)	10 (17.5%)	196.7 (123.6)	643.7 (108.4)	18.5 (12.3)
Off	n = 53	45 (84.9%)	43.3 (11.5)	30.8 (8.1)	43 (81.1%)	2 (3.8%)	3 (5.7%)	8 (15.1%)	40 (75.5%)	13 (24.5%)	171.0 (128.1)	639.0 (88.8)	22.7 (16.8)

**Table 3:** Changes in rSST, Total sedentary time and MVPA by intervention version

	rSST (min/day)			Sedentary Time (min/day)			MVPA (min/day)		
	Baseline	Week 16	Difference	Baseline	Week 16	Difference	Baseline	Week 16	Difference
OVERALL	184.7 (6.1)	99.8 (7.3)	−84.8 (−103.6, −66.1)	635.8 (5.7)	635.3 (7.4)	−0.4 (−18.7, 17.8)	18.6 (0.8)	16.1 (1.1)	−2.5 (−5.1, 0.2)
LOCKOUT, EARN & TEXT	194.1 (16.1)	68.5 (17.2)	−125.7 (−172.0, −79.3)	672.7 (14.5)	674.8 (15.9)	2.1 (−40.0, 44.3)	14.2 (2.1)	14.4 (2.2)	0.3 (−5.8, 6.3)
LOCKOUT & EARN	210.0 (20.3)	115.7 (24.2)	−94.3 (−156.2, −32.4)	634.9 (18.9)	691.3 (26.6)	56.4 (−4.8, 117.5)	8.6 (2.6)	10.0 (3.9)	1.5 (−7.6, 10.6)
LOCKOUT & TEXT	178.1 (16.1)	105.3 (19.9)	−72.8 (−122.9, −22.6)	633.6 (14.8)	635.2 (18.7)	1.5 (−45.2, 48.3)	32.1 (2.1)	16.6 (2.8)	−15.6 (−22.4, −8.8)
LOCKOUT	175.3 (19.0)	72.6 (21.2)	−102.7 (−161.5, −43.8)	608.9 (17.9)	636.8 (19.6)	27.9 (−22.9, 78.7)	14.6 (2.5)	15.6 (2.9)	1.0 (−6.2, 8.2)
TEXT & EARN	118.9 (15.9)	101.9 (17.7)	−17.0 (−63.6, 29.6)	628.2 (14.6)	613.3 (16.5)	−14.9 (−58.2, 28.4)	22.6 (15.9)	23.8 (2.3)	1.2 (−4.9, 7.3)
EARN	240.9 (14.9)	122.9 (17.4)	−118.1 (−163.0, −73.1)	615.1 (13.7)	606.9 (15.8)	−8.2 (−49.2, 32.8)	19.3 (1.9)	18.6 (2.3)	−0.7 (−6.7, 5.2)
TEXT	143.0 (16.5)	82.9 (21.3)	−60.1 (−113.0, −7.2)	622.3 (15.2)	583.7 (19.7)	−38.6 (−87.4, 10.3)	16.2 (2.2)	16.1 (2.9)	−0.1 (−7.2, 6.9)
CORE ONLY	216.9 (18.7)	128.6 (23.4)	−88.2 (−147.7, −28.8)	670.6 (17.3)	640.9 (22.4)	−29.7 (−84.5, 25.1)	21.1 (2.5)	13.9 (3.2)	−7.2 (−14.8, 0.4)

Note baseline and week 16 values are mean (SE) from mixed model. Differences are mean (95% confidence interval).

**Table 4:** Feasibility, User Burden, and Acceptability Outcomes

			LOCKOUT		TEXT		EARN	
		Overall	On	Off	On	Off	On	Off
**Feasibility**	Randomized (N)	110	55	55	59	51	57	53
	Study-related withdraw (N[%])	11 (10%)	7 (12.7%)	4 (7.3%)	6 (10.2%)	5 (9.8%)	2 (3.5%)	8 (15.1%)
	Non-study related withdraw (N[%])	5 (4.5%)	1 (1.8%)	4 (7.3%)	2 (3.4%)	3 (5.9%)	3 (5.3%)	2 (3.8%)
	Missing 16-week assessment (N[%])	14 (12.7%)	9 (16.4%)	5 (9.1%)	7 (11.9%)	7 (13.7%)	8 (14.0%)	6 (11.3%)
	Completed 16-week assessment (N[%])	80 (72.8%)	38 (69.1%)	42 (76.4.%)	44 (74.6%)	36 (70.6%)	44 (77.2%)	37 (69.8%)
**User burden**	Total Score, M(SD)	7.2 (6.5)	7.6 (6.3)	6.8 (6.8)	8.1 (6.6)	6.1 (6.4)	7.9 (6.3)	6.3 (6.8)
	Difficulty of Use, M(SD)	0.8 (0.6)	0.8 (0.6)	0.7 (0.7)	0.9 (0.7)	0.7 (0.6)	0.9 (0.7)	0.7 (0.6)
	Physical, M(SD)	0.1 (0.2)	0.1 (0.2)	0.1 (0.2)	0.1 (0.2)	0.1 (0.2)	0.1 (0.1)	0.1 (0.2)
	Time and Social, M(SD)	0.3 (0.4)	0.3 (0.5)	0.3 (0.4)	0.4 (0.5)	0.3 (0.3)	0.3 (0.4)	0.3 (0.4)
	Mental and Emotional, M(SD)	0.4 (0.6)	0.4 (0.5)	0.4 (0.6)	0.5 (0.5)	0.3 (0.6)	0.5 (0.5)	0.3 (0.6)
	Privacy, M(SD)	0.3 (0.6)	0.3 (0.7)	0.3 (0.6)	0.4 (0.7)	0.3 (0.6)	0.3 (0.6)	0.3 (0.6)
	Financial, M(SD)	0.01 (0.1)	0.03 (0.2)	0.0 (0.0)	0.02 (0.2)	0.0 (0.0)	0.02 (0.2)	0.0 (0.0)
		N = 79	N = 37	N = 42	N = 43	N = 36	N = 44	N = 35
Acceptability		% satisfied/very satisfied or % yes						
Overall Satisfaction with StandupTV app (% satisfied)		55%	57.2	52.6	48.5	60.6	51.4	58.6
Overall, was the StandUPTV app helpful in reducing your recreational sedentary screen time? (% yes)		94%	100	89.5	90.3	96.97	89.3	97.2
If it were made available, would you be interested in continuing the use of the StandUPTV app after the study is over? (% yes)		78%	74.1	80.6	80.7	75	77.1	78.6
Would you download an app like this to your smartphone? (% yes)		85%	77.8	89.5	84.4	85.9	81.1	89.3
Would you recommend the StandUPTV app to other people? (% yes)		92%	91.9	92.6	90.3	93.9	89.2	96.3
Proportion using app at least 1/day (% yes)		59%	70.4	51.4	64.5	54.5	61.1	57.2
Satisfaction with technical support		85%	74.6	92.1	84.8	84.8	89.2	79.3
Responsiveness of study staff		99%	100	97.4	100	97	97.3	100
		N = 66	N = 28	N = 38	N = 33	N = 33	N = 37	N = 29

## Data Availability

The data that support the findings of this study have some privacy restrictions applied and are not publicly available. The data are, however, available from the authors upon reasonable request and with the permission of Arizona State University.

## Abbreviations

MVPA: moderate to vigorous physical activity
BMI: body mass index
rSST: recreational sedentary screen time
TV: television viewing

## References

[1] Cardoso PC, Caldeira TCM, Sousa TM, Claro RM. Changes in Screen Time in Brazil: A Time-Series Analysis 2016–2021. Am J Health Promot. 2023;37(5):681–4. 36651005 10.1177/08901171231152147

[2] Harvey DL, Milton K, Jones AP, Atkin AJ. International trends in screen-based behaviours from 2012 to 2019. Prev Med. 2022;154:106909. 34871663 10.1016/j.ypmed.2021.106909

[3] Bertuol C, da Silveira MHC, Krug RR, Kupske JW, Mielke GI, Del Duca GF. Use of electronic devices in leisure time modifies the prevalence and factors associated with sedentary behavior derived exclusively from excessive television viewing among Brazilian adults. BMC Public Health. 2023;23(1):1602. 37608246 10.1186/s12889-023-16517-7 PMC10463304

[4] Runacres A, Mackintosh KA, Knight RL, Sheeran L, Thatcher R, Shelley J, Impact of the COVID-19 Pandemic on Sedentary Time and Behaviour in Children and Adults: A Systematic Review and Meta-Analysis. Int J Environ Res Public Health. 2021;18(21). 10.3390/ijerph182111286 PMC8583678 34769800

[5] Trott M, Driscoll R, Irlado E, Pardhan S. Changes and correlates of screen time in adults and children during the COVID-19 pandemic: A systematic review and meta-analysis. EClinicalMedicine. 2022;48:101452. 35615691 10.1016/j.eclinm.2022.101452 PMC9122783

[6] Liu S, Coulter R, Sui W, Nuss K, Rhodes RE. Determinants of recreational screen time behavior following the COVID-19 pandemic among Canadian adults. Appl Physiol Nutr Metab. 2023;48(8):595–602. 37037046 10.1139/apnm-2022-0379

[7] Walton-Pattison E, Dombrowski SU, Presseau J. ‘Just one more episode’: Frequency and theoretical correlates of television binge watching. J Health Psychol. 2018;23(1):17–24. 27106091 10.1177/1359105316643379

[8] Healy GN, Dunstan DW, Salmon J, Cerin E, Shaw JE, Zimmet PZ, Breaks in sedentary time: beneficial associations with metabolic risk. Diabetes care. 2008;31:661–6. 18252901 10.2337/dc07-2046

[9] Grace MS, Dillon F, Barr ELM, Keadle SK, Owen N, Dunstan DW. Television Viewing Time and Inflammatory-Related Mortality. Med Sci Sports Exerc. 2017;49(10):2040–7. 28514265 10.1249/MSS.0000000000001317

[10] Grontved A, Hu FB. Television viewing and risk of type 2 diabetes, cardiovascular disease, and all-cause mortality: a meta-analysis. JAMA. 2011;305(23):2448–55. 21673296 10.1001/jama.2011.812 PMC4324728

[11] Keadle SK, Moore SC, Sampson JN, Xiao Q, Albanes D, Matthews CE. Causes of Death Associated With Prolonged TV Viewing: NIH-AARP Diet and Health Study. Am J Prev Med. 2015;49(6):811–21. 26215832 10.1016/j.amepre.2015.05.023 PMC4656060

[12] Schmid D, Leitzmann MF. Television viewing and time spent sedentary in relation to cancer risk: a meta-analysis. J Natl Cancer Inst. 2014;106(7). 10.1093/jnci/dju098 24935969

[13] Sun JW, Zhao LG, Yang Y, Ma X, Wang YY, Xiang YB. Association Between Television Viewing Time and All-Cause Mortality: A Meta-Analysis of Cohort Studies. Am J Epidemiol. 2015;182(11):908–16. 26568572 10.1093/aje/kwv164

[14] Takagi H, Hari Y, Nakashima K, Kuno T, Ando T, Group A. Meta-analysis of the Relation of Television-Viewing Time and Cardiovascular Disease. Am J Cardiol. 2019;124(11):1674–83. 31586528 10.1016/j.amjcard.2019.08.032

[15] Shi H, Hu FB, Huang T, Schernhammer ES, Willett WC, Sun Q, Sedentary Behaviors, Light-Intensity Physical Activity, and Healthy Aging. JAMA Netw Open. 2024;7(6):e2416300. 38861256 10.1001/jamanetworkopen.2024.16300 PMC11167497

[16] Whitaker KM, Buman MP, Odegaard AO, Carpenter KC, Jacobs DR Jr., Sidney S, Sedentary Behaviors and Cardiometabolic Risk: An Isotemporal Substitution Analysis. Am J Epidemiol. 2018;187(2):181–9. 28595346 10.1093/aje/kwx209 PMC5860012

[17] Ekelund U, Steene-Johannessen J, Brown WJ, Fagerland MW, Owen N, Powell KE, Does physical activity attenuate, or even eliminate, the detrimental association of sitting time with mortality? A harmonised meta-analysis of data from more than 1 million men and women. Lancet. 2016;388(10051):1302–10. 27475271 10.1016/S0140-6736(16)30370-1

[18] Christensen MA, Bettencourt L, Kaye L, Moturu ST, Nguyen KT, Olgin JE, Direct Measurements of Smartphone Screen-Time: Relationships with Demographics and Sleep. PLoS One. 2016;11(11):e0165331. 27829040 10.1371/journal.pone.0165331 PMC5102460

[19] Augner C, Vlasak T, Aichhorn W, Barth A. The association between problematic smartphone use and symptoms of anxiety and depression-a meta-analysis. J Public Health (Oxf). 2023;45(1):193–201. 34585243 10.1093/pubmed/fdab350

[20] Li L, Zhang Q, Zhu L, Zeng G, Huang H, Zhuge J, Screen time and depression risk: A meta-analysis of cohort studies. Front Psychiatry. 2022;13:1058572. 36620668 10.3389/fpsyt.2022.1058572 PMC9815119

[21] Ramsey Buchanan L, Rooks-Peck CR, Finnie RKC, Wethington HR, Jacob V, Fulton JE, Reducing Recreational Sedentary Screen Time: A Community Guide Systematic Review. Am J Prev Med. 2016;50(3):402–15. 26897342 10.1016/j.amepre.2015.09.030 PMC9664246

[22] Otten JJ, Jones KE, Littenberg B, Harvey-Berino J. Effects of television viewing reduction on energy intake and expenditure in overweight and obese adults: a randomized controlled trial. Arch Intern Med. 2009;169(22):2109–15. 20008695 10.1001/archinternmed.2009.430

[23] Raynor HA, Steeves EA, Bassett DR Jr., Thompson DL, Gorin AA, Bond DS. Reducing TV watching during adult obesity treatment: two pilot randomized controlled trials. Behav Ther. 2013;44(4):674–85. 24094792 10.1016/j.beth.2013.04.012

[24] Spring B, Pellegrini C, McFadden HG, Pfammatter AF, Stump TK, Siddique J, Multicomponent mHealth Intervention for Large, Sustained Change in Multiple Diet and Activity Risk Behaviors: The Make Better Choices 2 Randomized Controlled Trial. J Med Internet Res. 2018;20(6):e10528. 29921561 10.2196/10528 PMC6030572

[25] Nguyen P, Le LK, Nguyen D, Gao L, Dunstan DW, Moodie M. The effectiveness of sedentary behaviour interventions on sitting time and screen time in children and adults: an umbrella review of systematic reviews. Int J Behav Nutr Phys Act. 2020;17(1):117. 32958052 10.1186/s12966-020-01009-3 PMC7504841

[26] Collins LM. Optimization of Behavioral, Biobehavioral, and Biomedical Interventions: The Multiphase Optimization Strategy (MOST). Springer International Publishing; 2018.

[27] Keadle S, Hasanaj K, Leonard-Corzo K, Tolas A, Crosley-Lyons R, Pfisterer B, StandUPTV: Preparation and optimization phases of a mHealth intervention to reduce sedentary screen time in adults. Contemp Clin Trials. 2024;136:107402. 38000452 10.1016/j.cct.2023.107402 PMC10922360

[28] Kiernan M, Schoffman DE, Lee K, Brown SD, Fair JM, Perri MG, The Stanford Leisure-Time Activity Categorical Item (L-Cat): a single categorical item sensitive to physical activity changes in overweight/obese women. Int J Obes (Lond). 2013;37(12):1597–602. 23588625 10.1038/ijo.2013.36 PMC4731089

[29] Harris PA, Taylor R, Thielke R, Payne J, Gonzalez N, Conde JG. Research electronic data capture (REDCap)--a metadata-driven methodology and workflow process for providing translational research informatics support. J Biomed Inform. 2009;42(2):377–81. 18929686 10.1016/j.jbi.2008.08.010 PMC2700030

[30] Lawrence CE, Dunkel L, McEver M, Israel T, Taylor R, Chiriboga G, A REDCap-based model for electronic consent (eConsent): Moving toward a more personalized consent. J Clin Transl Sci. 2020;4(4):345–53. 33244416 10.1017/cts.2020.30 PMC7681162

[31] Cleland CM. REDCap for MOST 2018 [Available from: https://cadio.org/redcap-with-most/.

[32] Suh H, Shahriaree N, Hekler EB, Kientz JA. Developing and Validating the User Burden Scale: A Tool for Assessing User Burden in Computing Systems. New York, NY, USA: Association for Computing Machinery; 2016.

[33] Dziak JJ, Collins LM, Wagner AT. Factorial Power Plan SAS Macro Users’ Guide Version 1.0. 2013.

[34] van Buuren S, Groothuis-Oudshoorn K. mice: Multivariate Imputation by Chained Equations in R. Journal of Statistical Software. 2011;45(3):1–67.

[35] Deng Y, Chang C, Ido MS, Long Q. Multiple Imputation for General Missing Data Patterns in the Presence of High-dimensional Data. Sci Rep. 2016;6:21689. 26868061 10.1038/srep21689 PMC4751511

[36] Rubin DB. Multiple Imputation for Nonresponse in Surveys. New York, NY: John Wiley & Sons In; 1987.

[37] Lewinski AA, Crowley MJ, Miller C, Bosworth HB, Jackson GL, Steinhauser K, Applied Rapid Qualitative Analysis to Develop a Contextually Appropriate Intervention and Increase the Likelihood of Uptake. Med Care. 2021;59(Suppl 3):S242–S51. 33976073 10.1097/MLR.0000000000001553 PMC8132894

[38] Piercy KL, Troiano RP, Ballard RM, Carlson SA, Fulton JE, Galuska DA, The Physical Activity Guidelines for Americans. JAMA. 2018;320(19):2020–8. 30418471 10.1001/jama.2018.14854 PMC9582631

[39] Smith DM, Duque L, Huffman JC, Healy BC, Celano CM. Text Message Interventions for Physical Activity: A Systematic Review and Meta-Analysis. Am J Prev Med. 2020;58(1):142–51. 31759805 10.1016/j.amepre.2019.08.014 PMC6956854

[40] Wu J, Brunke-Reese D, Lagoa CM, Conroy DE. Assessing the impact of message relevance and frequency on physical activity change: A secondary data analysis from the random AIM trial. Digit Health. 2024;10:20552076241255656. 10.1177/20552076241255656 PMC11113026 38784050

[41] Murtagh EM, Murphy MH, Milton K, Roberts NW, O’Gorman CS, Foster C. Interventions outside the workplace for reducing sedentary behaviour in adults under 60 years of age. Cochrane Database Syst Rev. 2020;7(7):CD012554. 32678471 10.1002/14651858.CD012554.pub2 PMC7389819

